# 

**Published:** 1962

**Authors:** 


### Contents

| | | |
|---|---|---|
| CASUALTY SURGEON | *H. K. Bourns* | 69 |
| SURVEY OF A PSYCHOGERIATRIC WARD | *D. M. D. White* | 78 |
| ACUTE ENCEPHALITIS | *Clinico-Pathological Conference* | 81 |
| OBITUARY | | 88 |
| NOTES AND NEWS | | 89 |
| EXAMINATION RESULTS | | 89 |
| APPOINTMENTS | | 90 |
| PROGRAMMES OF MEDICAL SOCIETIES | | 91 |

